# Thoracoabdominal endometriosis mimicking pulmonary tuberculosis in a TB-endemic setting: a case report

**DOI:** 10.1186/s13256-026-06150-4

**Published:** 2026-06-14

**Authors:** Murtaza Lamuwalla, Rukhsaar Ali, Joshua Ngimbwa, Caroline Ngimba, Mahmoud Arafa, Blessing Mathew, Mandela Makakala

**Affiliations:** 1 Family Medicine Resident, The Agakhan University, East Africa Medical College, Dar-es-Salaam, Tanzania; 2 Department of Family Medicine, The Aga Khan Hospital, Dar-es-Salaam, Tanzania; 3 Internal Medicine resident, The Agakhan University, East Africa Medical College, Dar-es-Salaam, Tanzania; 4 Department of Internal Medicine, The Aga Khan Hospital, Dar-es-Salaam, Tanzania; 5 Department of Pathology, The Aga Khan Hospital, Dar-es-Salaam, Tanzania; 6 Department of Radiology, The Aga Khan Hospital, Dar es Salaam, Tanzania; 7 Department of Surgery, The Aga Khan Hospital, Dar es Salaam, Tanzania

**Keywords:** Case report, Thoracic endometriosis, Mesenteric endometriosis, Pulmonary tuberculosis mimic, Hydropneumothorax

## Abstract

**Background:**

Endometriosis is a common gynecologic condition, but extra-pelvic manifestations are rare and often pose significant diagnostic challenges. Thoracic and abdominal endometriosis may present with nonspecific respiratory and abdominal symptoms that closely mimic pulmonary tuberculosis, particularly in TB-endemic regions, leading to delayed or incorrect diagnosis.

**Case presentation:**

We report the case of a 40-year-old Tanzanian woman with a previous history of treated pulmonary tuberculosis who presented with a 6-month history of progressive shortness of breath, right-sided chest pain, and abdominal distension. Imaging revealed a massive right hydropneumothorax with near-total lung collapse and ascites, initially raising concern for post-tuberculous disease. Pleural fluid cytology demonstrated a hemorrhagic effusion. Diagnostic laparoscopy revealed hemorrhagic ascites, mesenteric nodular lesions, and extensive uterine adhesions. Histopathological examination of the mesenteric nodules confirmed endometriosis. The patient was treated with gonadotropin-releasing hormone agonist therapy (goserelin) and underwent pleurodesis, with subsequent clinical improvement and resolution of symptoms.

**Conclusion:**

This case highlights thoracoabdominal endometriosis as an important differential diagnosis in women of reproductive age presenting with unexplained respiratory symptoms in TB-endemic settings. Awareness of this rare entity is essential to avoid misdiagnosis and unnecessary anti-tuberculous therapy. Early consideration of extra-pelvic endometriosis and timely histological confirmation can facilitate appropriate management and improve patient outcomes.

## Introduction

Endometriosis is a common gynecologic disorder, affecting an estimated 6–13% of women of reproductive age [1]. It is defined by the presence of functional endometrial glands and stroma outside the uterine cavity. Although it most frequently involves pelvic organs such as the ovaries, uterosacral ligaments, and peritoneum, it can also manifest at extra-pelvic sites, including the abdominal wall particularly in surgical scars following cesarean section or laparotomy and very rarely within the lungs or pleura [2]. Thoracic involvement is estimated to occur in approximately 2.5–5.6% of extra-pelvic endometriosis cases [3].

Clinical presentation varies depending on the site involved. Pulmonary endometriosis may present with catamenial hemoptysis, cyclical cough, pleuritic chest pain or recurrent hemothorax, or pneumothorax temporally related to menses. However, some patients may remain asymptomatic with incidental nodules detected on imaging [4]. In contrast, abdominal wall endometriosis typically presents as a painful or enlarging mass near a previous surgical scar that becomes more tender or prominent during menstruation. Less commonly, patients may present with cyclical abdominal pain without a palpable mass or with umbilical bleeding or discharge [5].

Diagnosis of pulmonary or abdominal wall endometriosis is often challenging due to the rarity of these locations, the nonspecific nature of symptoms, and variability in imaging findings. Imaging modalities such as ultrasound, computed tomography (CT), and magnetic resonance imaging (MRI) may demonstrate hemorrhagic or cystic nodules near surgical scars in abdominal wall disease and lung nodules, infiltrates, or lesions that fluctuate with the menstrual cycle in pulmonary disease. However, definitive diagnosis typically requires histopathological confirmation through biopsy, surgical excision, or video-assisted thoracoscopic surgery (VATS) [6].

Management most often involves surgical resection of visible lesions particularly in abdominal wall disease and/or hormonal therapy aimed at suppressing ectopic endometrial tissue. Although data on imaging follow-up after medical therapy in pulmonary endometriosis remain limited, isolated reports have demonstrated regression of diaphragmatic or pulmonary lesions with corresponding symptom improvement after approximately six months of hormonal treatment as observed on follow-up MRI [7].

In tuberculosis endemic regions, tuberculous pleural effusion remains the most common cause of pleural disease and frequently presents with non-specific clinical features, thereby complicating diagnosis and contributing to a diagnostic bias toward tuberculosis. Conventional pleural fluid analysis has important limitations, often necessitating the use of adjunctive modalities. Thoracic ultrasound plays a crucial role in the stepwise evaluation of pleural effusions by identifying features such as septations and loculations, while medical thoracoscopy is considered a gold-standard diagnostic tool as it allows direct visualization and targeted pleural biopsy, significantly improving diagnostic accuracy. An integrated diagnostic approach combining thoracic ultrasound and thoracoscopy has therefore been proposed to optimize diagnostic yield. In this context, rare conditions such as thoracic endometriosis may be overlooked. However, it should be considered in women of reproductive age presenting with pleural effusion and chest pain, particularly when clinical “red flags,” such as cyclical symptoms, are present [8, 9].

Given the high prevalence of pulmonary tuberculosis in our setting, clinicians may be inclined to attribute chronic or recurrent respiratory symptoms to TB. Nevertheless, in women of reproductive age presenting with long-standing or cyclical respiratory complaints especially hemoptysis, pleuritic chest pain, or unexplained pulmonary nodules, extra-pelvic endometriosis should be considered in the differential diagnosis alongside infectious causes. Early recognition of this rare entity is essential to prevent misdiagnosis and to facilitate timely tissue confirmation and appropriate surgical or hormonal management.

## Case presentation

A 40-year-old Tanzanian woman (Para 0 + 2, living 0) with no known comorbidities had a significant past medical history of pulmonary tuberculosis treated in 2011, a medically managed miscarriage in 2021, a myomectomy in 2021, and an ectopic pregnancy treated with methotrexate in 2025. Menstrual cycles were regular but associated with significant dysmenorrhea.

The patient presented with a 6-month history of progressively worsening shortness of breath and abdominal pain. The dyspnea was gradual in onset and associated with right-sided and non-radiating chest pain without accompanying cough, fever, or constitutional symptoms. In addition, there was progressive abdominal pain with distension and weight loss without changes in bowel habits, urinary symptoms, or abnormal vaginal discharge.

On examination, the patient was alert and afebrile with stable vital signs (BP 102/66 mmHg, RR 22 breaths/min, HR 85 beats/min, SpO₂ 96% on room air, and temperature 36.7 °C). Respiratory examination revealed no use of accessory muscles with dullness to percussion and reduced air entry accompanied by crepitations over the right mammary and inframammary regions. Abdominal examination demonstrated a symmetrically distended abdomen that moved with respiration, generalized tenderness, and audible bowel sounds. The patient was admitted for further evaluation.

**Fig. 1:**
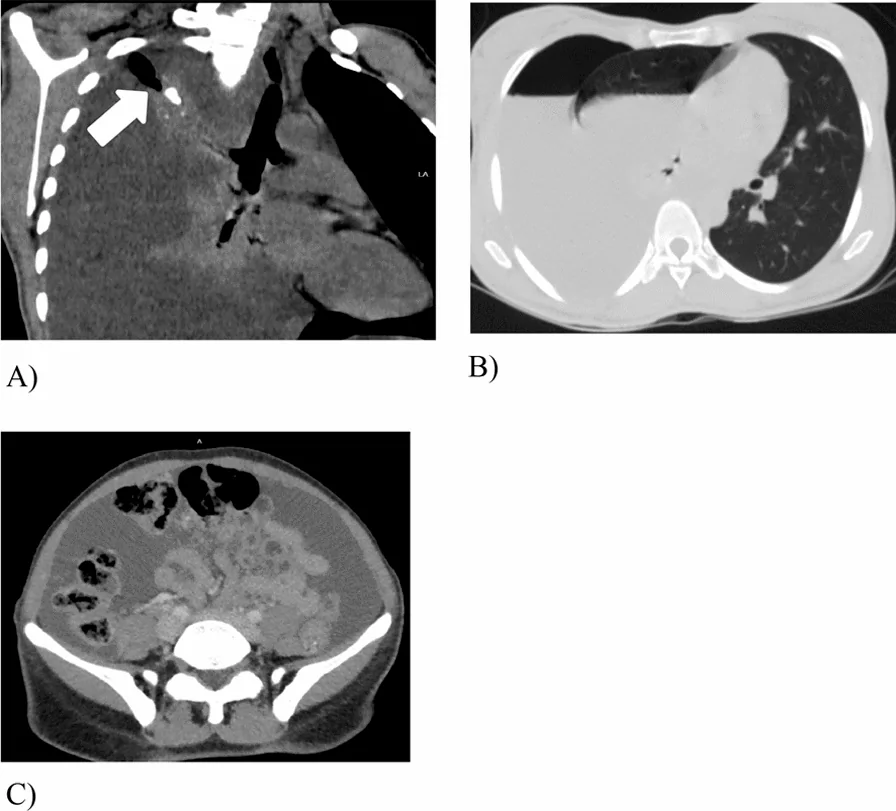
CT scan of the chest and abdomen. **A**- Chest coronal oblique view images showing broncho-pleural fistula, **B**- Chest axial view showing marked hydro-pneumothorax and **C**- Abdomen axial view showing abdominal ascites

Computed tomography (CT) of the chest (Fig. 1A and B) demonstrated a massive right-sided hydropneumothorax with near-total collapse of the right lung and a suspected bronchopleural fistula. CT imaging of the abdomen and pelvis (Fig. 1C) revealed moderate ascites and multiple uterine fibroids. A chest tube was inserted, draining hemorrhagic fluid which was sent for analysis along with other laboratory investigations (Table 1).

**Table 1:** Key hematological, biochemical, microbiological, pleural fluid, ascitic fluid, and tumor marker results obtained during evaluation

Laboratory test	Result	Reference
CBC		
WBC	4.78 × 10^9^/L	4.0–11.0 × 10^9^/L
HB	8.2 g/dl	12.3–15.3 g/dl
PLT	334 × 10^9^/L	150–450 × 10^9^/L
Sputum		
Gram stain	No organism seen	
GeneXpert	MTB not detected	
Acid fast bacilli	No acid-fast bacilli seen	
Culture and sensitivity	No bacterial growth	
Serum (ADA) adenosine Deaminase	9.1 U/L	0–15 U/L
Serum creatinine	44.2 umol/L	59–104 umol/L
INR	0.9 s	0.9–1.15 s
PT	9 s	11.5–15 s
aPPT	30.7 s	24.9–36.8 s
ESR	17.2 mm/h	0–20 mm/h
Pleural fluid		
Gram stain	No organism seen	
GeneXpert	MTB not detected	
Cytology	Hemorrhagic effusion with subacute inflammation	
Culture and sensitivity	No bacterial/fungal growth	
Ascitic fluid		
Gram stain	No organism seen	
GeneXpert	MTB not detected	
Culture and sensitivity	No bacterial/fungal growth	
CA-125	113.4 U/ml	0–35.0 U/ml
CA19.9	7.63 U/ml	0–37 U/ml
CEA	0.77 ng/ml	3.8–5 ng/ml
Beta HCG	0.10 mIU/ml	Normal menstruating < 5.3

ADA: adenosine deaminase; AFB: acid-fast bacilli; aPTT: activated partial thromboplastin time; CA-125: cancer antigen 125; CA19-9: carbohydrate antigen 19–9; CBC: complete blood count; CEA: carcinoembryonic antigen; ESR: erythrocyte sedimentation rate; Hb: hemoglobin; INR: international normalized ratio; MTB: Mycobacterium tuberculosis; PLT: platelet; PT: prothrombin time; WBC: white blood cell

**Fig. 2:**
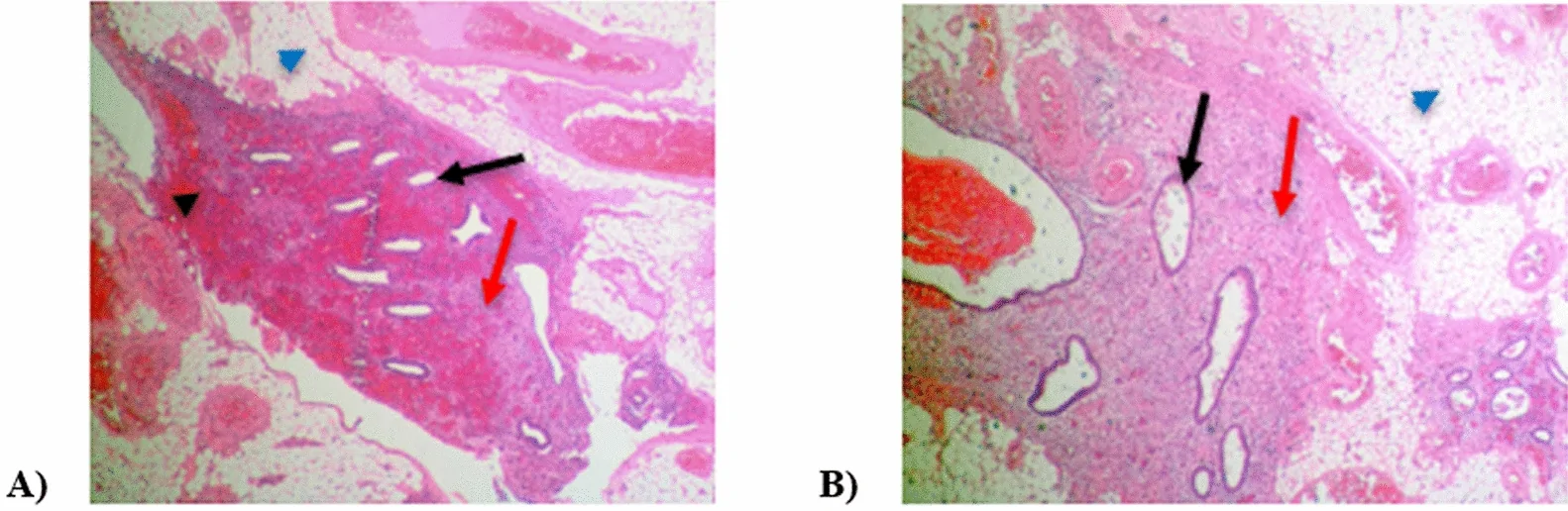
**A** (X4) and **B** (X10) H/E sections showing nests of endometrial glands (black arrow) and stroma (red arrow) embedded within mesenteric fatty tissue (blue arrowhead). Hemorrhage is noted (black arrowhead)

In view of the elevated CA-125 level and clinical findings, a gynecological consultation was obtained and thoracoabdominal endometriosis was suspected. Exploratory diagnostic laparoscopy was performed under general anesthesia. Intraoperatively, hemorrhagic ascites was encountered, together with multiple small bluish nodular deposits on the mesenteric fatty tissue of the small bowel, and dense adhesions between the uterus and the anterior abdominal wall. Representative nodules were biopsied laparoscopically with hemostasis achieved and sent for histopathological examination (Fig. 2) which confirmed endometriosis.

Bronchoscopy and thoracoscopy were recommended to further evaluate the suspected bronchopleural fistula and underlying pleural pathology; however, these were not performed due to financial constraints. Consequently, the diagnosis of bronchopleural fistula was based on radiological findings on CT imaging and was not confirmed bronchoscopically.

Medical therapy with a gonadotropin-releasing hormone (GnRH) agonist (goserelin) was initiated. The patient was managed conservatively with tube thoracostomy, with the chest tube remaining in situ for eight days and draining approximately 300–700 mL of hemorrhagic fluid daily. Pleurodesis was subsequently performed to prevent recurrence of the hydropneumothorax. The patient showed clinical improvement and was discharged in stable condition (BP 116/70 mmHg, RR 18 breaths/min, HR 77 beats/min, SpO_2_ 99% on room air, and temperature 36.6 °C).

At follow-up, there was significant symptomatic improvement, although reduced air entry persisted on examination without crepitations. Vital signs remained stable (BP 123/75 mmHg, RR 16 breaths/min, HR 72 beats/min, SpO_2_ 99% on room air, and temperature 36.8 °C). Follow-up imaging, including a chest radiograph to assess radiological resolution and evaluate the status of the suspected bronchopleural fistula, was advised but was not performed due to financial limitations. Therefore, follow-up assessment was based on clinical improvement rather than imaging findings.

## Discussion

This case illustrates a rare presentation of thoracoabdominal endometriosis with concurrent pulmonary and intra-abdominal involvement mimicking pulmonary tuberculosis in a TB-endemic setting. The clinical presentation of progressive dyspnea, right-sided chest pain, and abdominal distension over several months is nonspecific and may easily be attributed to chronic pulmonary infections or sequelae of prior tuberculosis particularly in regions such as Tanzania where tuberculosis remains highly prevalent [10, 11].

Thoracic endometriosis is the most common form of extra-pelvic endometriosis although it remains an uncommon entity overall. It is encompassed within thoracic endometriosis syndrome (TES) which includes catamenial pneumothorax, hemothorax, hemoptysis, and pulmonary nodules. The presence of a massive right-sided hydropneumothorax with near-complete lung collapse and a suspected bronchopleural fistula is consistent with pleural involvement. The predominance of right-sided disease aligns with established theories involving transdiaphragmatic migration of endometrial tissue through diaphragmatic defects facilitated by clockwise peritoneal fluid circulation [12].

The pathogenesis of thoracoabdominal endometriosis is multifactorial. Proposed mechanisms include retrograde menstruation with transcoelomic spread, coelomic metaplasia, and lymphatic or hematogenous dissemination [10, 12]. The coexistence of mesenteric nodules and uterine adhesions in this case supports a combination of transperitoneal and lymphovascular spread. In addition, prior gynecological surgery such as myomectomy may contribute to iatrogenic implantation of endometrial tissue onto peritoneal or mesenteric surfaces [5, 6].

Simultaneous thoracic and intra-abdominal involvement is particularly rare and highlights the systemic potential of endometriosis. Such widespread disease may arise from chronic pelvic endometriosis with transdiaphragmatic extension or through lymphatic dissemination [7, 10]. Clinically these patients may present with nonspecific respiratory and abdominal symptoms which can mimic tuberculosis, malignancy or other chronic inflammatory conditions often resulting in delayed diagnosis [7].

Radiological and cytological findings further complicate diagnosis. Imaging in this case demonstrated hydropneumothorax and ascites which was initially suggestive of a post-tuberculous process. However, the presence of hemorrhagic pleural effusion and negative infectious workup prompted further investigation. Definitive diagnosis was established through histopathological identification of endometrial glands and stroma within mesenteric nodules. These findings are consistent with existing literature which emphasizes that tissue diagnosis remains essential due to the nonspecific nature of imaging findings [6, 7].

Management of thoracoabdominal endometriosis requires a multidisciplinary approach. Hormonal suppression remains the cornerstone of therapy aiming to induce a hypoestrogenic state and promote regression of ectopic endometrial tissue. Treatment with a GnRH agonist (goserelin) resulted in clinical improvement and symptom stabilization. Hormonal therapy is particularly valuable in cases of diffuse or unresectable disease. In addition, pleurodesis was performed to prevent recurrence of hydropneumothorax which is a recognized intervention in thoracic endometriosis with pleural involvement [12].

The favorable clinical outcome characterized by improvement in respiratory and abdominal symptoms underscores the effectiveness of combined medical and procedural management. Long-term follow-up is essential due to the risk of recurrence following cessation of hormonal therapy. Maintenance therapy with continuous oral contraceptives or progestins may be considered to sustain disease suppression [10].

This case highlights several important clinical considerations. First, in tuberculosis-endemic settings alternative diagnoses should be considered in women of reproductive age presenting with unexplained respiratory symptoms particularly when features are atypical for tuberculosis. Second, endometriosis should be recognized as a multisystem disease capable of involving both thoracic and abdominal compartments. Finally, early suspicion and histological confirmation are crucial in guiding appropriate management and avoiding unnecessary anti-TB treatment.

This report has important limitations. First, although thoracic endometriosis was strongly suspected based on the right-sided hemorrhagic hydropneumothorax, negative infectious workup, concomitant histologically confirmed mesenteric endometriosis, and clinical improvement after hormonal therapy and pleurodesis, thoracic histological confirmation was not obtained. Bronchoscopy and thoracoscopy with pleural or bronchial biopsy were recommended but were not performed because of financial constraints. Therefore, the diagnosis of thoracic involvement remains clinicoradiological rather than histologically proven. Second, the suspected bronchopleural fistula was based on CT findings and was not confirmed bronchoscopically. Third, no follow-up imaging was available to objectively document radiological resolution after treatment. These limitations reduce the certainty with which thoracic endometriosis and bronchopleural fistula can be definitively established; however, the abdominal component of endometriosis was confirmed histologically.

## Conclusion

Thoracoabdominal endometriosis is a rare but important mimic of pulmonary tuberculosis in women of reproductive age, particularly in TB-endemic settings. This case demonstrates the value of maintaining a broad differential diagnosis when respiratory symptoms, hemorrhagic effusion, ascites, and negative infectious workup coexist. Histopathology confirmed mesenteric endometriosis, while thoracic involvement was suspected clinicoradiologically. Early recognition may prevent unnecessary anti-tuberculous therapy and allow timely hormonal and procedural management.

## Data Availability

All data generated or analyzed during this study are included in this published article. Additional details are available from the corresponding author upon reasonable request.

## Abbreviations

aPPT: Activated partial prothrombin time
BP: Blood pressure
CT scan: Computed tomography scan
CBC: Complete blood count
HB: Hemoglobin
HR: Heart rate
INR: International normalized ratio
MRI: Magnetic resonance imaging
PT: Prothrombin time
PLT: Platelets
RR: Respiratory rate
SPO_2_: Oxygen saturation
TB: Tuberculosis
WBC: White blood cell
